# Neuromuscular exercise for chronic musculoskeletal pain in older people: a randomised controlled trial in primary care in Hong Kong

**DOI:** 10.3399/bjgp20X714053

**Published:** 2021-01-26

**Authors:** Regina Wing Shan Sit, Shirley Yue Kwan Choi, Bo Wang, Dicken Cheong Chun Chan, Dexing Zhang, Benjamin Hon Kei Yip, Samuel Yeung Shan Wong

**Affiliations:** Division of Family Medicine and Primary Health Care, Jockey Club School of Public Health and Primary Care, Faculty of Medicine, the Chinese University of Hong Kong, Hong Kong.; Department of Family Medicine, the New Territories East Cluster, Hospital Authority, Hong Kong, China.; Division of Family Medicine and Primary Health Care, Jockey Club School of Public Health and Primary Care, Faculty of Medicine, the Chinese University of Hong Kong, Hong Kong.; Division of Family Medicine and Primary Health Care, Jockey Club School of Public Health and Primary Care, Faculty of Medicine, the Chinese University of Hong Kong, Hong Kong.; Division of Family Medicine and Primary Health Care, Jockey Club School of Public Health and Primary Care, Faculty of Medicine, the Chinese University of Hong Kong, Hong Kong.; Division of Family Medicine and Primary Health Care, Jockey Club School of Public Health and Primary Care, Faculty of Medicine, the Chinese University of Hong Kong, Hong Kong.; Division of Family Medicine and Primary Health Care, Jockey Club School of Public Health and Primary Care, Faculty of Medicine, the Chinese University of Hong Kong, Hong Kong.

**Keywords:** aged, chronic musculoskeletal pain, exercise therapy, neuromuscular exercise, pain, randomised controlled trial

## Abstract

**Background:**

Exercise therapy is commonly prescribed by primary care physicians (PCPs) in the management of chronic musculoskeletal (MSK) pain.

**Aim:**

To evaluate the clinical effectiveness of a supervised neuromuscular (NM) exercise programme in older people with chronic MSK pain.

**Design and setting:**

This was a 12-week, two-arm, randomised controlled trial comparing 6 weeks of supervised NM exercise versus waiting list controls. The authors enrolled 72 participants with chronic MSK pain at seven public primary care clinics.

**Method:**

Participants were randomly allocated in block sizes of 12 to the NM (*n* = 36) and control groups (*n* = 36) in a 1:1 ratio. Data were collected at baseline, 6, and 12 weeks. The primary outcome was the Brief Pain Inventory (BPI) pain severity score at 6 weeks post-intervention. Secondary outcomes included the BPI interference score; Pain Self-Efficacy Questionnaire (PSEQ), Short Form Health Survey (SF-12), 7-item Generalised Anxiety Disorder (GAD-7), and 9-item Patient Health Questionnaire (PHQ-9) scores; and functional measurements using the Timed-Up- and-Go test and handgrip strength.

**Results:**

At 6 weeks, the NM group demonstrated a significantly greater improvement in the BPI pain severity score (between-group difference = −1.27; 95% confidence interval [CI] = −2.08 to −0.45; *P*<0.01), PSEQ (between-group difference = 6.5; 95% CI = 2.22 to 10.77; *P*<0.01), and SF-12 physical scores (between-group difference = 3.4; 95% CI = 0.05 to 6.75; *P*<0.05) compared with the control group. Statistically significant overall trends of improvement were also observed for the BPI interference and PHQ-9 scores.

**Conclusion:**

NM exercise has the potential to reduce pain and improve self-efficacy and physical function in older people with chronic MSK pain. It can be an option for PCPs in exercise prescriptions.

## INTRODUCTION

Chronic musculoskeletal (MSK) pain is very common among older patients, with varying impacts on functional, psychological, and social impairment.^1^ According to the World Health Organization’s 2015 report, MSK health conditions represent a global threat to the healthy ageing population, with extensive adverse effects.^2^ Painful MSK conditions are strongly associated with a reduced capacity to engage in physical activity, resulting in functional decline, frailty, reduced wellbeing, and loss of independence.^3^ In the Global Burden Disease Study 2017, MSK pain conditions were the highest contributor to global disability among all chronic diseases.^4^

Chronic MSK pain is commonly managed in primary care,^5^ and accounts for 15%–20% of all annual visits to GPs.^6^^,^^7^ Medication is the most common treatment modality, with one in five older adults (18%) regularly taking analgesics.^8^ Analgesic use is associated with significant side effects; its prescription and consumption are complicated in older people with multiple comorbidities, polypharmacy, and age- and frailty-related changes in pharmacokinetics and pharmacodynamics.^9^

Exercise therapy is a well-known non-pharmacological modality in chronic pain management.^10^ Neuromuscular (NM) exercise is one form of exercise therapy focused on improving sensorimotor control and attaining functional joint stabilisation. There are some differences between NM and conventional strength training. NM exercise incorporates strength training and also emphasises joint control and quality of movement. Participants are trained in functional exercises in various positions that resemble conditions of daily life and more strenuous activities. The strength training in NM exercise usually consists of non-weight-bearing exercises that train isolated muscles selectively, followed by weight-bearing exercises involving multiple joints. The quantity of muscle output is emphasised, and the level of training and progression is guided by the patient’s performance. The exercise protocol of NM is generally more complicated than strength training.^11^^,^^12^

The use of NM exercise has been advocated as a rehabilitation measure for sports injuries in athletic young adults.^13^ Recently, its role has been evaluated in knee and hip arthritis conditions, and it has been reported to be feasible and effective at reducing pain and improving function in hip and knee osteoarthritis.^11^^,^^14^^–^^16^ Moreover, it has been shown to improve articular cartilage quality, muscle activation pattern, and joint biomechanics.^17^^,^^18^ However, only a few randomised clinical trials have been conducted, and these have only assessed single joint diseases.^19^ Given the engagement of multiple joints and muscle groups in NM training for obtaining static and dynamic equilibrium of loaded segments, its therapeutic effect in pain management is believed to be general, rather than for a single joint pain or specific disease pathology.

The current study aimed to evaluate the clinical effectiveness of a supervised NM exercise programme in Chinese older people with chronic MSK pain.

**Table:** How this fits in

Neuromuscular (NM) exercise focuses on improving sensorimotor control and attaining functional joint stabilisation, and has been advocated as a rehabilitation measure for sports injuries in athletic young adults. Knowledge of its role in the management of chronic musculoskeletal (MSK) pain in older adults remains unknown. If proven to be clinically effective, it can therefore be used as an option for exercise prescriptions by primary care physicians. The aim of this trial was to evaluate if NM exercise reduces pain among older people with chronic MSK pain. This study found that a between-group difference of 1.27 was detected between the NM exercise and control groups, equivalent to a 12.7% improvement in the Brief Pain Inventory pain severity score, thus achieving the minimal clinically important difference of 10% improvement in chronic pain trials. This signified that NM exercise may be considered as one of the options in chronic pain management.

## METHOD

### Settings and participants

This was a 12-week, two-arm, parallel, randomised controlled trial (RCT). The authors enrolled patients from seven public primary care clinics in the New Territories East region of Hong Kong, and assessed them at a university-affiliated primary care clinic.

The eligibility criteria were screened by a trained research assistant and confirmed by primary care physicians. The inclusion criteria were: community-dwelling older adults aged ≥65 years; mobile >10 metres with or without a walking aid; presenting with chronic MSK pain over a consecutive 3-month period; pain intensity score ≥3 on a numerical rating scale of 10; stable baseline activity; and ability to understand written and verbal Chinese. The exclusion criteria were: participants with dementia or mild cognitive impairment; MSK pain due to inflammatory rheumatic disease; a history of stroke or major surgery in the previous 6 months; terminal illness; serious mental illness; severe or uncontrolled heart disease; major joint replacement or spinal surgery; comorbid conditions that might impede active participation in the study; and meeting the diagnostic criteria of chronic fatigue syndrome from the Centers for Disease Control and Prevention (CDC).^20^

### Randomisation, allocation concealment, and blinding

An off-site statistician performed block randomisation in block sizes of 12 to allocate the participants into two groups on a 1:1 ratio. The allocation sequence was concealed from the researcher and patients using sequentially numbered, opaque, sealed envelopes. The corresponding envelopes were opened at the time of intervention assignment once all of the enrolled participants had undergone all baseline assessments. It was not possible for physicians and patients to be blinded in this open-label study; however, research assistants and statisticians involved in data collection and analysis, respectively, were blinded to the allocation status.

### Intervention group with neuromuscular training

The authors applied the NM training principles reported by Clausen *et al*, which comprise the following key elements: functional performance; postural control; extremity muscle strength; balance; functional trunk and peripheral joint stability; and gait retraining.^21^ Exercises were mainly performed in closed kinetic chains in different positions (for example, lying, sitting, and standing) with the intention of obtaining low, evenly distributed articular surface pressure through muscular co-activation. The protocol was customised to address the physical limitations of older people, and was pilot tested using 10 participants and found to be safe and feasible.

Each participant underwent 60-minute supervised training sessions performed by a board-certified physical fitness instructor, twice a week for 6 weeks (12 sessions in total). Each session consisted of warming up (10 minutes), NM exercise (45 minutes), and cooling down (5 minutes). The warm-up exercise consisted of stretching tight and facilitated muscles. This was followed by the circuit NM training exercise; each exercise was performed in two sets of 15 repetitions. The cooling-down session involved general stretching. The group training did not involve machines or weight lifting. After 6 weeks of supervised NM training, all participants were encouraged to follow the instructions in the exercise pamphlet and continue home exercise (Supplementary Table S1).

### Control (waiting-list) group with exercise brochures

Participants in the waiting-list group were offered the same exercise classes after completion of the study. To minimise bias, the authors framed the intervention description in an expectancy-neutral manner. A generic exercise pamphlet for older people downloaded from the official government website was distributed to the participants for self-reading and practice during the study period.^22^

### The use of co-interventions

Participants were not restricted from seeking other interventions during the study period. The authors obtained records of the use of co-interventions from the clinical management system, an electronic system operated by the Hospital Authority in Hong Kong. In addition, the authors asked participants to recall their private treatment.

### Outcome measures

Trained research assistants blinded to the allocation conducted the face-to-face interviews at the study site to collect the outcomes using paper-based questionnaires. The authors collected data for all outcome measures at baseline and at 6- and 12-weeks post-intervention. The primary outcome was the post-intervention Brief Pain Inventory (BPI) pain severity score at 6 weeks. A timepoint of 6 weeks was chosen to evaluate the maximum effect of NM exercise in the supervised period. The BPI (score range 0–10) consists of 4-item severity and 7-item interference subscale scores, and is a validated tool for older adults with non-malignant pain.^23^ Secondary outcomes included the BPI interference score; the Pain Self-Efficacy Questionnaire (PSEQ, score range 0–60), which measures the participants’ confidence while performing 10 activities using a 7-point scale, with higher scores indicating stronger self-efficacy;^24^ and functional measurements using the Timed-Up-and-Go test (TUGT) and handgrip strength. The authors measured health-related quality of life using the physical and mental component scores of the Short Form Health Survey (SF-12, score range 0–100).^25^ The 9-item Patient Health Questionnaire (PHQ-9, score range 0–27) and the 7-item Generalised Anxiety Disorder (GAD-7, score range 0–21) were used to measure depression and anxiety, respectively.^26^^,^^27^ Only questionnaires with a validated Chinese version were used in the study. The objective functional measurements obtained were the TUGT^28^ and handgrip strength.^29^

The authors recorded patients’ demographics and body mass index (BMI), and used the FRAIL scale to screen for frailty (pre-frail 1–2; frail 3–5)^30^ and the SARC-F scale to screen for sarcopenia (≥4 indicates sarcopenia).^31^ Baseline physical activity level was assessed using the Physical Activity Scale for the Elderly (PASE),^32^ and the Stanford Expectations of Treatment Scale (SETS) questionnaire was used to detect bias of treatment expectation.^33^

### Safety monitoring

Standardisation forms were used to monitor and report side effects and adverse events. Any serious adverse events were reported to the ethics committee within 24 hours.

### Sample size calculation

At the time of sample size calculation, the authors did not identify any studies that evaluated the effectiveness of NM exercise on chronic pain with BPI pain score as outcome, nor did they identify studies that report the BPI pain mean score in the general population. Therefore, the authors estimated the effect size based on similar clinical trials, with land-based exercise as intervention and pain as the outcome. The effect sizes ranged approximately from 0.37 to 0.94.^34^^–^^36^ An average effect size of 0.70 was assumed. With alpha set at 0.05, a sample size of 72 with 36 participants in each group had 83% power to detect an effect size of 0.70 using a two-sample *t*-test.

### Statistical analysis

The authors used analysis of covariance to assess the intervention effects on both the primary and secondary outcomes at 6-weeks post-intervention following the intention-to-treat (ITT) principle, adjusting baseline BPI scores, sex, and BMI. Prior to ITT analysis, missing values were imputed using a multiple imputation model, in which 20 completed datasets were imputed using chain equations under the assumption that data were missing at random. The imputation model included: sex; age; BMI; BPI, PSEQ, SF-12, GAD-7, PHQ-9, FRAIL, SARC-F, and TUGT scores; handgrip strength; and SETS results. The authors combined the effect estimates based on Rubin’s rule,^37^ and used linear mixed models (LMM) for secondary analysis to assess significant changes over time in the 12-week study period, following the ITT principle. A non-linear relationship was observed over time for a number of outcomes; therefore, the authors treated time as a categorical variable to capture the non-linear relationship. The authors assumed the baseline outcomes in both groups were equal, as reflected by the intercept term. The treatment variable was not part of the model; however, its interaction with time remained in the model.^38^ LMM were used to examine the overall treatment effect of non-linear relationship, with time indicator 0 for baseline and 1 for follow-up visits. The statistical package IBM SPSS Statistics (version 21) and R (version 3.4.3) were used.

## RESULTS

**Figure 1.**
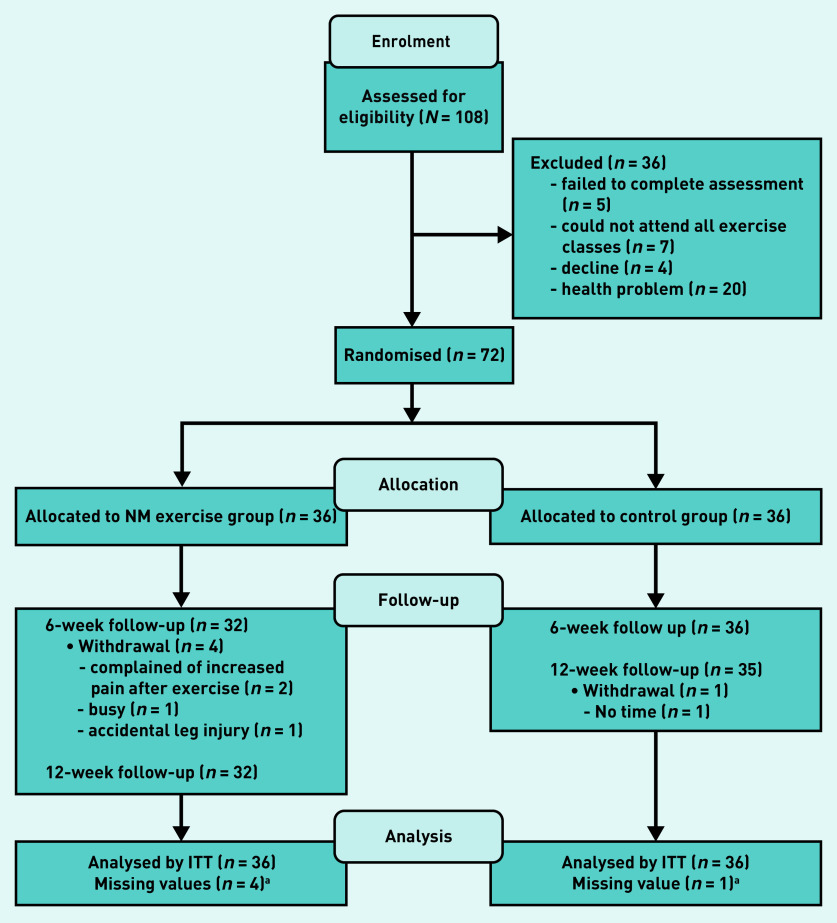
***Flow chart of patient recruitment and randomisation for study participation.*** ***^a^ Missing values were imputed for analysis. ITT = intention to treat. NM = neuromuscular.***

The study was conducted from June 2017 to April 2019. The researchers enrolled 72 of the 108 participants considered for trial inclusion based on the eligibility criteria, and randomly allocated them to two groups containing 36 participants each (Figure 1). All participants completed the baseline questionnaire and were included in the ITT analysis. The dropout rate was 11.1% (*n* = 4) in the NM group, and 2.8% (*n* = 1) in the control group. Participants in the NM group completed a median of 11 (interquartile range 10–12) exercise sessions.

The study sample (93.1% female) had a mean age of 70.3 years (standard deviation [SD] 4.7); 19.4% were overweight, of which 41.7% were obese; 77.8% were pre-frail; and 20.8% had sarcopenia. There was no between-group difference in the physical activity level and treatment expectancy (Table 1). About 93.0% of the participants reported multiple pain sites, with the most common sites being the knee (84.7%), back (59.7%), shoulder (50.0%), and neck (22.2%) (Supplementary Table S2).

**Table 1.** Baseline characteristics of participants

**Characteristic**	**Total, *n*= 72**	**NM exercise group, *n*= 36**	**CG, *n*= 36**
**Mean age, years (SD)**	70.3 (4.7)	70.8 (5.0)	69.8 (4.3)

**Sex, *n* (%)**			
Male	5 (6.9)	2 (5.6)	3 (8.3)
Female	67 (93.1)	34 (94.4)	33 (91.7)

**BMI, *n* (%)**			
<18.5 (underweight)	4 (5.6)	4 (11.1)	0 (0.0)
18.5–22.9 (normal)	24 (33.3)	14 (38.9)	10 (27.8)
23.0–24.9 (overweight)	14 (19.4)	5 (13.9)	9 (25.0)
≥25.0 (obese)	30 (41.7)	13 (36.1)	17 (47.2)

**Frailty, *n* (%)**			
Robust (= 0)	12 (16.7)	7 (19.4)	5 (13.9)
Pre-frailty (1–2)	56 (77.8)	27 (75.0)	29 (80.6)
Frailty (3–5)	4 (5.6)	2 (5.6)	2 (5.6)

**Sarcopenia, *n* (%)**			
Positive (≥4)	15 (20.8)	4 (11.1)	11 (30.6)
Negative (≤3)	57 (79.2)	32 (88.9)	25 (69.4)

**PSEQ, mean (SD)**	41.5 (10.5)	41.0 (11.0)	42.0 (10.1)

**BPI, mean (SD)**			
Severity	4.5 (1.6)	4.4 (1.5)	4.6 (1.7)
Interference	3.9 (2.2)	3.4 (2.3)	4.4 (2.0)

**SF-12, mean (SD)**			
Physical	45.6 (6.5)	46.8 (6.7)	44.5 (6.2)
Mental	44.1 (9.5)	45.1 (11.1)	43.0 (7.7)

**TUGT, mean (SD)**	12.9 (3.8)	12.7 (3.9)	12.9 (3.8)

**HGS, mean (SD)**			
Left	18.0 (4.6)	17.7 (4.0)	18.3 (5.1)
Right	18.8 (4.8)	18.7 (4.1)	18.9 (5.5)

**GAD-7, mean (SD)**	3.4 (4.1)	3.5 (4.9)	3.3 (3.1)

**PHQ-9, mean (SD)**	4.9 (3.5)	4.8 (4.2)	5.0 (2.7)

**PASE, mean (SD)**	106.6 (38.0)	109.9 (35.9)	103.2 (40.2)

**SETS, mean (SD)**			
Positive	2.7 (0.9)	2.7 (0.8)	2.6 (0.9)
Negative	5.7 (1.0)	5.5 (1.2)	5.9 (0.8)

BMI = body mass index. BPI = Brief Pain Inventory. CG = control group. GAD-7 = 7-item Generalised Anxiety Disorder. HGS = handgrip strength. NM = neuromuscular. PASE = Physical Activity Scale for the Elderly. PHQ-9 = 9-item Patient Health Questionnaire. PSEQ = Pain Self-Efficacy Questionnaire. SD = standard deviation. SETS = Stanford Expectations of Treatment Scale. SF-12 = 12-Item Short Form Health Survey. TUGT = Timed-Up-and-Go test.

At the primary endpoint of 6 weeks, between-group comparisons indicated significantly greater improvement in the BPI pain severity score in the NM group compared with the control group (between-group difference = −1.27; 95% confidence interval [CI] = −2.08 to −0.45; *P*<0.01), with a calculated effect size of 0.63. In addition, the NM group showed an improvement in the PSEQ (between-group difference = 6.5; 95% CI = 2.22 to 10.77; *P*<0.01) and SF-12 physical scores (between-group difference = 3.4; 95% CI = 0.05 to 6.75; *P*<0.05) (Table 2). The primary outcome of the BPI pain severity score remained statistically significant at 12 weeks (*P*<0.05) (Table 3).

**Table 2.** The results of group effect on primary and secondary outcomes at 6-weeks post-intervention using intention to treat and analysis of covariance

**Outcome variables**		**NM exercise group, *n*= 36, mean (SD)**	**CG, *n*= 36, mean (SD)**	**Model 1^a^**			**Model 2^b^**		

				**Mean difference between groups (95% CI)**	***P*-value**	**Cohen’s *d***	**Mean difference between groups (95% CI)**	***P*-value**	**Cohen’s *d***
**Primary outcome BPI (severity)**									
	Wk 0	4.4 (1.5)	4.6 (1.7)	—	—	—	—	—	—
	Wk 6	3.1 (2.1)	4.6 (1.9)	−1.31 (−2.08 to −0.54)	**<0.01**	0.66	−1.27 (−2.08 to −0.45)	**<0.01**	0.63

**Secondary outcomes BPI (interference)**									
	Wk 0	3.4 (2.3)	4.4 (2.0)	—	—	—	—	—	—
	Wk 6	2.5 (2.3)	4.0 (2.2)	−0.74 (−1.51 to 0.02)	0.06	0.35	−0.67 (−1.48 to 0.15)	0.11	0.29

**PSEQ**									
	Wk 0	41.3 (10.8)	42.0 (10.1)	—	—	—	—	—	—
	Wk 6	45.1 (9.2)	38.6 (10.7)	6.87 (2.84 to 10.89)	**<0.01**	0.70	6.50 (2.22 to 10.77)	**<0.01**	0.63

**SF-12**									
Physical	Wk 0	46.8 (6.7)	44.5 (6.2)	—	—	—	—	—	—
	Wk 6	48.0 (8.3)	43.4 (7.1)	3.15 (−0.01 to 6.31)	0.05	0.41	3.4 (0.05 to 6.75)	**<0.05**	0.46
Mental	Wk 0	45.1 (11.1)	43.0 (7.7)	—	—	—	—	—	—
	Wk 6	48.9 (9.7)	44.8 (10.1)	2.90 (−0.98 to 6.78)	0.14	0.29	3.07 (−1.08 to 7.23)	0.15	0.29

**TUGT**									
	Wk 0	12.7 (3.9)	13.0 (3.8)	—	—	—	—	—	—
	Wk 6	11.3 (2.9)	12.4 (2.7)	−0.98 (−2.16 to 0.21)	0.10	0.41	−1.01 (−2.25 to 0.23)	0.11	0.41

**HGS**									
Left	Wk 0	17.7 (4.0)	18.3 (5.1)	—	—	—	—	—	—
	Wk 6	19.7 (6.0)	18.7 (5.5)	1.41 (−0.77 to 3.60)	0.20	0.29	1.40 (−0.95 to 3.74)	0.24	0.35
Right	Wk 0	18.7 (4.1)	18.9 (5.5)	—	—	—	—	—	—
	Wk 6	19.8 (5.0)	19.7 (5.1)	0.19 (−1.65 to 2.02)	0.63	0.11	0.15 (−1.90 to 2.21)	0.57	0.14

**GAD-7**									
	Wk 0	3.5 (4.9)	3.3 (3.1)	—	—	—	—	—	—
	Wk 6	5.3 (9.6)	4.9 (3.5)	0.08 (−2.79 to 2.94)	0.85	0.06	0.11 (−2.94 to 3.16)	0.86	0.06

**PHQ-9**									
	Wk 0	4.8 (4.2)	5.0 (2.7)	—	—	—	—	—	—
	Wk 6	4.5 (4.01)	6.0 (3.5)	−1.34 (−2.89 to 0.21)	0.10	0.35	−1.11 (−2.76 to 0.54)	0.20	0.29

a Adjusting for baseline score.

b Adjusting for baseline score, sex, and BMI. Bold data indicates statistically significant. BPI = Brief Pain Inventory. CG = control group. CI= confidence interval. GAD-7 = 7-item Generalised Anxiety Disorder. HGS = handgrip strength. ITT = intention to treat. NM = neuromuscular. PHQ-9 = 9-item Patient Health Questionnaire. PSEQ = Pain Self-Efficacy Questionnaire. SD = standard deviation. SF-12 = 12-Item Short Form Health Survey. TUGT = Timed-Up-and-Go test. Wk = week.

**Table 3.** The results of group effect on primary and secondary outcomes at 12 weeks post-intervention using intention to treat and analysis of covariance

**Outcome variables**		**NM exercise group, *n*= 36 mean (SD)**	**CG, *n*= 36, mean (SD)**	**Model 1^a^**			**Model 2^b^**		

				**Mean difference between groups (95% CI)**	***P*-value**	**Cohen’s *d***	**Mean difference between groups (95% CI)**	***P*-value**	**Cohen’s *d***
**Primary outcome BPI (severity)**									
	Wk 0	4.4 (1.5)	4.6 (1.7)	—	—	—	—	—	—
	Wk 12	2.7 (2.2)	4.1 (1.9)	−1.27 (−2.13 to −0.41)	**<0.01**	0.65	−1.28 (−2.18 to −0.38)	**<0.01**	0.63

**Secondary outcomes BPI (interference)**									
	Wk 0	3.4 (2.3)	4.4 (2.0)	—	—	—	—	—	—
	Wk 12	2.3 (2.3)	3.2 (2.2)	−0.43 (−1.33 to 0.47)	0.35	0.20	−0.19 (−1.13 to 0.75)	0.70	0.09

**PSEQ**									
	Wk 0	41.3 (10.8)	42.0 (10.1)	—	—	—	—	—	—
	Wk 12	44.7 (9.7)	41.0 (9.0)	3.97 (0.11 to 7.83)	**<0.05**	0.46	3.70 (−0.38 to 7.78)	0.08	0.41

**SF-12**									
Physical	Wk 0	46.8 (6.7)	44.5 (6.2)	—	—	—	—	—	—
	Wk 12	51.1 (8.8)	46.1 (8.3)	3.70 (−0.04 to 7.44)	0.05	0.46	3.61 (−0.39 to 7.61)	0.08	0.46
Mental	Wk 0	45.1 (11.1)	43.0 (7.7)	—	—	—	—	—	—
	Wk 12	48.1 (9.3)	46.3 (8.3)	0.97 (−2.75 to 4.69)	0.62	0.13	0.37 (−3.59 to 4.33)	0.80	0.06

**TUGT**									
	Wk 0	12.7 (3.9)	13.0 (3.8)	—	—	—	—	—	—
	Wk 12	11.1 (2.8)	11.8 (2.5)	−0.55 (−1.63 to 0.53)	0.33	0.20	−0.41 (−1.59 to 0.77)	0.50	0.18

**HGS**									
Left	Wk 0	17.7 (4.0)	18.3 (5.1)	—	—	—	—	—	—
	Wk 12	20.3 (15.9)	22.2 (16.4)	−1.44 (−8.85 to 5.97)	0.61	0.16	−0.90 (−9.03 to 7.23)	0.59	0.16
Right	Wk 0	18.7 (4.1)	18.9 (5.5)	—	—	—	—	—	—
	Wk 12	19.6 (5.3)	19.8 (4.3)	−0.09 (−1.97 to 1.79)	0.67	0.11	−0.12 (−2.14 to 1.90)	0.61	0.14

**GAD-7**									
	Wk 0	3.5 (4.9)	3.3 (3.1)	—	—	—	—	—	—
	Wk 12	4.3 (4.9)	4.5 (3.2)	−0.32 (−2.04 to 1.40)	0.72	0.09	−0.14 (−1.96 to 1.68)	0.86	0.06

**PHQ-9**									
	Wk 0	4.8 (4.2)	5.0 (2.7)	—	—	—	—	—	—
	Wk 12	5.3 (4.9)	5.7 (3.5)	−0.29 (−2.05 to 1.47)	0.73	0.09	−0.15 (−2.01 to 1.71)	0.78	0.06

a Adjusting for baseline score.

b Adjusting for baseline score, sex, and BMI. Bold data indicates statistically significant. BMI = body mass index. BPI = Brief Pain Inventory. CG = control group. CI= confidence interval. GAD-7 = 7-item Generalised Anxiety Disorder. HGS = handgrip strength. ITT = intention to treat. NM = neuromuscular. PHQ-9 = 9-item Patient Health Questionnaire. PSEQ = Pain Self-Efficacy Questionnaire. SD = standard deviation. SF-12 = 12-Item Short Form Health Survey. TUGT = Timed-Up-and-Go test. Wk = week.

**Figure 2.**
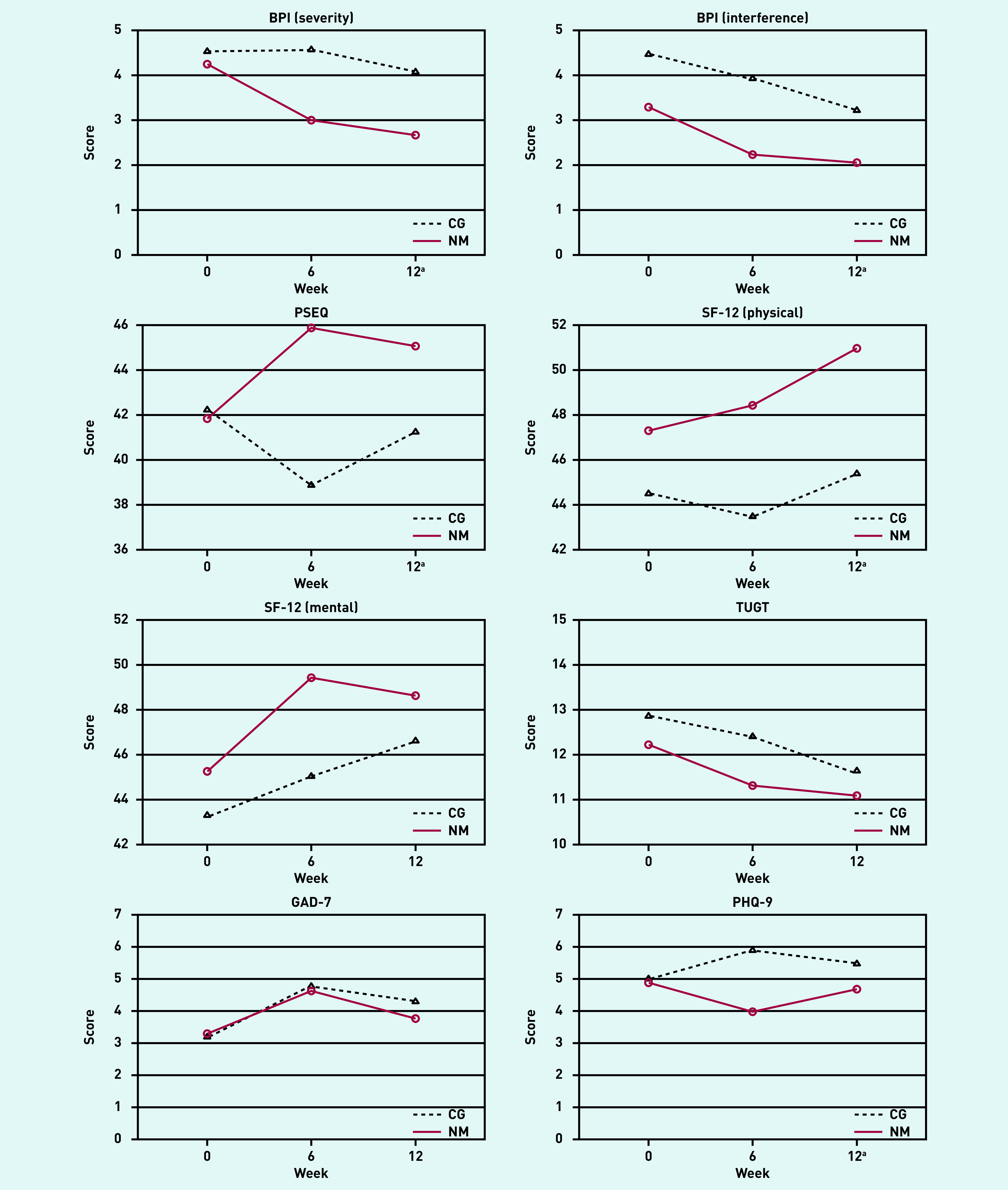
***Changes in BPI, PSEQ, SF-12, TUGT, GAD-7, and PHQ-9 scores from baseline to 12 weeks follow-up.*** ***^a^Significant change effect (*P**<***0.05) over time in the 12-week study period. BPI = Brief Pain Inventory. CG = control group. GAD-7 = 7-item Generalised Anxiety Disorder. NM = neuromuscular. PHQ-9 = 9-item Patient Health Questionnaire. PSEQ = Pain Self-Efficacy Questionnaire. SF-12 = 12-item Short Form Health Survey. TUGT = Timed-Up-and-Go test.***

The authors observed statistically significant overall trends of improvement in BPI pain severity score, BPI interference score, PSEQ and PHQ-9 scores, and SF-12 physical scores throughout the 12-week study period (Figure 2 and Supplementary Table S3). No related adverse events were reported.

## DISCUSSION

### Summary

The authors’ findings indicate that NM exercise could potentially reduce pain and improve self-efficacy and physical function in older people with chronic MSK pain. A 12.7% improvement in BPI pain severity scores compared with the control group at 6 weeks was found, with sustained effect to 12 weeks, achieving the minimal clinically important difference (MCID) of 10% improvement in chronic pain trials.^39^ The pain-reducing effect of NM exercise was further confirmed in the secondary analyses. The improvement of PSEQ score in the NM group was statistically significant at 6 weeks, but not at 12 weeks. This suggested that a coach-supervised NM exercise may be important in enhancing the confidence of pain management in older people, through encouragement, motivation, support, and problem solving.^40^^,^^41^ The improvement of SF-12 physical score in the NM group compared with the control group also exceeded the reported MCID of 3.29 at 6 weeks.^42^ Although the improvement of BPI interference score and PHQ-9 score were not statistically significant in the primary analysis, the overall trends of improvement were positive and statistically significant in the secondary analysis.

### Strengths and limitations

To the best of the authors’ knowledge, this is the first RCT to assess the general effect of NM exercise on chronic MSK pain in older people using guideline-recommended core outcome measures for chronic pain trials.^43^ A low dropout rate and high treatment protocol adherence was observed. The study has several limitations. First, the nocebo effects in the waiting list control group could have led to a response bias.^44^ However, it is generally accepted that a waiting list group might be an appropriate choice for testing whether a novel intervention could be useful.^45^ Furthermore, the researchers attempted to minimise this bias by balancing the baseline expectations regarding treatment effects^33^ and measuring the objective outcomes.^46^ Second, the study duration was only 3 months; therefore, the long-term efficacy on pain intensity of this intervention remains unclear. Third, participants were all community-dwelling older people; therefore, their baseline pain score, functional level, and psychological status were generally better than those who are not in a community setting, thus limiting the generalisability of the findings to older people in nursing homes or residential care settings.

### Comparison with existing literature

The authors observed reduced pain in the NM group, which is consistent with the previously reported overall decreasing pain trajectory for hip and knee osteoarthritis after NM exercise.^14^^–^^16^ The effect sizes of NM exercise on pain were 0.63 at both 6 and 12 weeks, which is consistent with a recent Cochrane review of land-based exercise interventions for chronic MSK pain, including aerobic exercise, resistance, and balance coordination training, with effect sizes ranging from 0.38 to 0.93.^47^ However, direct comparison is difficult due to differences in study design, interventions, and control groups. One of the advantages of using NM exercise for pain management is that its dosing approach is according to individual performance and relatively pain-free motion; this is important for older participants because pain impedes regular exercise participation^48^ and adherence.^49^ The additional benefits of NM exercise are on proprioception and subsequent local and global functional stability; these were indirectly reflected in the statistically significant improvements in the SF-12 physical component scores. Although the objective measurement of function by the TUGT was not statistically significant, the positive trend of improvement may suggest that a larger sample size may be able to detect the treatment effect. The general benefits that active exercise participation has on happiness, through the release of endorphins, serotonins, dopamine, and other reward chemicals,^50^ and those of enjoyable social contacts with peers,^51^ were demonstrated in the overall trend of improvement in the depression status of the NM group.

### Implications for research and practice

Chronic MSK pain is commonly encountered and managed in primary care; however, it is often difficult for primary care physicians to prescribe the best kind of exercise therapy for their patients. These findings demonstrate the potential of NM exercise as an effective modality in chronic MSK management, especially in an older population with a high prevalence of pain at multiple sites. Future larger-scale RCTs are needed to compare the overall effectiveness of NM exercise with other exercise therapies.

NM exercises can be applied by medical personnel and allied health workers, such as physiotherapists, rehabilitation nurses, and physical fitness trainers involved in MSK pain management at different levels. These personnel could undergo structured NM training to learn the overall concept and techniques, and earn certification to teach the exercise in their own settings. Digital technologies, such as mobile apps, can be designed to disseminate information regarding the exercise protocol. The findings also demonstrate the possibility of implementing an evidence-based, low-cost intervention for chronic MSK pain management.
